# Increased levels of interleukin 31 (IL-31) in osteoporosis

**DOI:** 10.1186/s12865-015-0125-9

**Published:** 2015-10-08

**Authors:** Lia Ginaldi, Massimo De Martinis, Fedra Ciccarelli, Salvatore Saitta, Selene Imbesi, Carmen Mannucci, Sebastiano Gangemi

**Affiliations:** Department of Life, Health, & Environmental Sciences, University of L’Aquila, L’Aquila, Italy; Department of Clinical and Experimental Medicine, University of Messina, Messina, Italy

**Keywords:** Osteoporosis, Aging, IL-31, Inflammation, Translational medicine

## Abstract

**Background:**

Several inflammatory cytokines play a key part in the induction of osteoporosis. Until now, involvement of the Th2 cytokine interleukin-31 (IL-31) in osteoporosis hadn’t yet been studied. IL-31 is a proinflammatory cytokine mediating multiple immune functions, whose involvement in a wide range of diseases, such as atopic dermatitis, inflammatory bowel diseases and cutaneous lymphomas, is now emerging. Given the important role of IL-31 in inflammation, we measured its serum levels in postmenopausal osteoporotic patients.

**Methods and results:**

In fifty-six postmenopausal females with osteoporosis and 26 healthy controls, bone mineral density (BMD) measurements were performed by using calcaneal quantitative ultrasound (QUS) technique, confirmed at the lumbar spine and hip by dual energy X-ray absorptiometry (DXA). Both patients and controls were divided according to age (less or more than 65 years) and disease severity (T-score levels and presence of fractures). Serum IL-31 levels were measured by ELISA technique. Osteoporotic patients exhibited elevated levels of serum IL-31 compared with healthy controls (43.12 ± 6.97 vs 29.58 ± 6.09 pg/ml; *p* < 0.049). IL-31 expression was higher in over 65 years old patients compared to age-matched controls (45 ± 11.05 vs. 17.92 ± 5.92; *p* < 0.01), whereas in younger subjects no statistically significant differences were detected between patients and controls (37.91 ± 6.9 vs 32.08 ± 8.2). No statistically significant differences were found between IL-31 levels in patients affected by mild (T-score > -3) compared to severe (T-score < -3) osteoporosis (59.17 ± 9.22 vs 37.91 ± 10.52), neither between fractured and unfractured osteoporotic women (33.75 ± 9.16 vs 51.25 ± 8.9).

**Conclusions:**

We showed for the first time an increase of IL-31 serum levels in postmenopausal women with decreased BMD. Although they did not reflect the severity of osteoporosis and/or the presence of fractures, they clearly correlated with age, as reflected by the higher levels of this cytokine in aged patients.

## Background

In the last few years, many studies have been conducted to understand the processes that regulate physiological and pathological bone turnover and there was increasing evidence of a relationship between the immune system and bone [1, 2]. There is a mutual influence between the immune system and bone tissue cells, mediated by shared receptors, soluble molecules and signalling pathways [3]. T cells have been recognized as key regulators of osteoclast and osteoblast activity in different diseases able to induce osteoporosis, such as rheumatic diseases, bone metastasis, periodontitis and chronic infections [4–6]. Also paraphysiological conditions, such as aging and menopause, are associated to dysregulation of bone remodelling leading to loss of bone mass and consequentely, to a state of full-blown osteoporosis [7–9].

Osteoporosis is a systemic skeletal disease characterized by decreased bone mass and microarchitectural deterioration of bone tissue. An excessive bone resorption and/or an inadequate bone formation results in a subsequent increase in bone fragility and susceptibility to fractures [10]. The main regulator of osteoclast differentiation is the receptor activator of nuclear factor-kB (NF-kB) ligand (RANKL), a factor produced by osteoblasts/stromal cells and belonging to the tumour necrosis factor (TNF) family. In the presence of the growth factor M-CSF (macrophage-colony stimulating factor), the activation of its receptor RANK on the surface of osteoclast precursors and mature osteoclasts represents an essential signal for osteoclast differentiation and activation and consequentely bone resorption [11]. Interestingly, immune cells, mainly activated T lymphocytes and antigen presenting cells, can also express RANKL, therefore influencing bone remodelling, both directely and through cytokine production [12]. For example, IL-6, IL-17, TNF-α and IFN-γ promote bone loss by favouring osteoclast production and inhibiting osteoblast differentiation [13, 14]. Other cytokines, such as IL-4, IL-12 and IL-33, are strong suppressors of osteoclast differentiation and inhibit bone loss [15, 16]. In addition, many of these cytokines have pleiotropic functions and their role in bone remodelling is cell-type and concentration-dependent and also influenced by the variable cytokine network involved in different physiological and pathological conditions [3].

Interleukin-31 (IL-31) is a cytokine known for almost a decade, belonging to the gp130/IL-6 cytokine family [17] and preferentially expressed by activated memory CD45RO+ T lymphocytes skewed toward a Th2 phenotype [18–22]. Elucidation of the biological activities of IL-31 has only just begun and a biological role of IL-31 in immunity and inflammation is now emerging [18, 19]. It has been observed that this cytokine plays a predominant function in the mediation of inflammatory and lymphoma-associated itch [23], and a secondary one in the suppression of ongoing TH2 responses [17]. IL-31 signals through a heteromeric receptor complex composed of the IL-31 receptor alpha (IL-31RA) and the oncostatin M receptor beta (OSMR) subunits [20]. High receptor expression levels have been found in tissues involved in reproduction, in progenitor cells of myelomonocytic lineage, spleen, thymus, in addition to skin, lung, gut and other tissues [17, 20, 23, 24]. IL-31RA is also expressed by a small subpopulation of neurons, representing a critical neuro-immune link between Th2 cells and sensory nerves for the generation of immune-mediated itch [25]. The broad spectrum of its receptor expression on immune- and non-immune cells, suggests that this novel cytokine may have multiple, pleiotropic physiological functions, including regulating hematopoiesis and immune response, causing inflammation [17, 21].

IL-31 stimulates secretion of proinflammatory cytokines, chemokines, and matrix metalloproteinases [17, 26]. Moreover, it has been observed that IL-31 can positively and negatively affect Th1 and Th17 differentiation in an APC-free system [17]. These data suggest a potential function for IL-31 in regulating immune responses through modulating antigen-presenting cells or more directly T cell themselves. Earlier studies have shown involvement of IL-31 and its receptor components IL-31RA and OSMR in atopic dermatitis, pruritus and Th2-weighted inflammation [20, 27]. Until today it has been implicated in cutaneous pathologies [18, 20, 23, 28], inflammatory bowel diseases [29], atopy [18, 23, 27, 28, 30, 31], respiratory inflammation [32] and some types of tumours [33, 34], but its role in osteoporosis hadn’t yet been studied. The aim of our study was to evaluate the possible involvement of IL-31 also in subjects affected by osteoporosis.

## Methods

### Patients

Fifty-six post-menopausal females with osteoporosis (mean age ± standard deviation: 65.39 ± 9.68 years) and 26 age-matched (61.84 ± 8.03 years) healthy post-menopausal women as controls, were enrolled in this study after written informed consent. The study was approved by the local ethics committee and carried out in compliance with the Helsinky Declaration [35]. In all subjects, bone mineral density (BMD) measurements were performed by using computerized methods: calcaneal quantitative ultrasound (QUS) technique (Lunar Achilles Express densitometer), confirmed at the lumbar spine and hip by dual energy X-ray absorptiometry (DXA) (Hologic QDR 4500 W machine). BMD values obtained by DXA technique were expressed as T-score, i.e. the difference (number of standard deviations) between the BMD value of the examined subject and the mean value in the healthy reference population. Z-score (the patient’s BMD compared with the mean BMD of people of the same sex and age) and BMD values expressed as g/cm2, were also collected and they are shown in Table 1.

**Table 1:** Clinical and anthropometric features of patients (*n* = 56) and controls (*n* = 26). Data are expressed as Mean +/- SD (min; max)

	Patients	Controls
Age (years)	65 +/- 9 (39; 80)	62 +/- 8 (48; 76)
Height (cm)	153 +/- 0.06 (135; 165)	152 +/- 0.05 (142; 159)
Weight (kg)	63.83 +/- 9.43 (51; 86)	64.73 +/- 11.06 (51; 83)
Body mass index (kg/m^2^)	26.88 +/- 4.49 (19.34; 34.78)	27.70 +/- 3.55 (22.5; 34.58)
Total hip BMD (g/cm^2^)*	0.701 +/- 0.07 (0.460; 0.790)	0.990 +/- 0.10 (0.890; 1.300)
Total hip T-score*	−3.0 +/- 0.7 (-5.4; -2.0)	−0.1 +/- 1.0 (-1.1; 3.0)
Total hip Z-score*	−1.1 +/- 0.9 (-3.3; 0.2)	1.4 +/- 1.1 (-0.5; 4.0)
Calcanear QUS T-score *	−3.6 +/- 0.92 (-5.5; -1.79)	−0.73 +/- 1.26 (-2.3; 2.5)
ESR (mm/h)*	19.25 +/- 8.69 (6; 44)	12 +/- 7.56 (2; 27)
CRP (mg/L)*	5.75 +/- 6.60 (0.1; 41)	2.67 +/- 3.65 (0.1; 19)
BAP (μg/L)	17.68 +/- 6.57 (8.2; 30.8)	15.26 +/- 7.00 (5; 30.8)
CTX (pg/mL)*	380 +/- 246 (0.32; 900)	155 +/- 236 (5; 568))
PTH (pg/mL)	67.03 +/- 22.59 (28.6; 147)	66.04 +/- 15.20 (42.5; 88)

Legend: erythrocyte sedimentation rate (ESR); C-reactive protein (CRP); bone alkaline phosphatase (BAP)

; C-telopeptide cross-linked collagen type 1 (CTX); parathyroid hormone (PTH)

*Statistically significant difference between patients and controls (*p* < 0.001)

T-score values between +1 and -1 were considered normal, whereas T-score below -2.5 defined osteoporosis. Collected data also included careful medical history, accurate clinical examination, assessment of anthropometric parameters (height, weight and body mass index) and laboratory and instrumental tests, such as spinal X-ray and vertebral morphometry to check for any asymptomatic osteoporotic fractures. Among laboratory tests, we included inflammation markers, such as erythrocyte sedimentation rate (ESR) and C-reactive protein (CRP), as well as parathyroid hormone (PTH) values and serum markers of bone remodeling, such as bone alkaline phosphatase (BAP) and C-telopeptide cross-linked collagen type 1 (CTX).

None of the studied subjects had pathologies causing secondary osteoporosis, or took drugs that could affect bone metabolism [6]. Diagnosis was made according to the current criteria of the World Health Organization [10], which is based on the T-score for the classification of the BMD into categories of normal, osteopenia, osteoporosis, and severe osteoporosis. Thus patients were further divided, according to the BMD value, into two groups: 27 patients with T-score > -3 (mean age 64.59 ± 9.24 years) and 29 with T-score < -3 (mean age 66.13 ± 10.18 years). The definition of mild or severe osteoporosis is related to hip BMD values obtained from DXA. Both patients and controls were also divided according to age: less than or equal to 65 years (n. 43; mean age 56.91 ± 6.03 years) and older than 65 years (n. 39; mean age 72.38 ± 3.89 years). Serum IL-31 levels were measured in both patients and controls. Blood samples were obtained by venipuncture and centrifuged at 3000 rpm for 5 min. Serum samples were stored at -20 °C until use.

### IL-31 dosage

IL-31 protein levels were evaluated using a standard sandwich ELISA kit according to the manufacturer’s recommendations (USCN LIFE SCIENCE, Houston, TX, USA).

The adsorbance was measured at 450 nm by a microspectrophotometer mod. 340 ATTC (SLT Lab. Instruments Salzburg, Austria). Data were expressed as pg/ml.

As reported by the manufacturer, the minimum detectable dose of IL 31 is less than 5.5 pg/ml. The value of 15.6 is referred to the lower point IL 31 standard curve. Moreover the Lower Limit of Detection (LLD) has been determined as follow: (Mean negative control optical density) + 2* (StDev of negative control optical density), as suggested by manufacturer. Our LLD was 1.9 pg/ml. In addition, to better detect IL-31 concentration in our samples, we performed a standard curve starting from 7.8 pg/ml.

### Statistical analysis

Data were expressed as medians ± SEM. Differences between two unpaired groups were analyzed by Mann–Whitney test. The statistical analysis was performed with SPSS for Windows (version 17.0). The level of statistical significance was always set at P < 0.05.

## Results

**Fig. 1:**
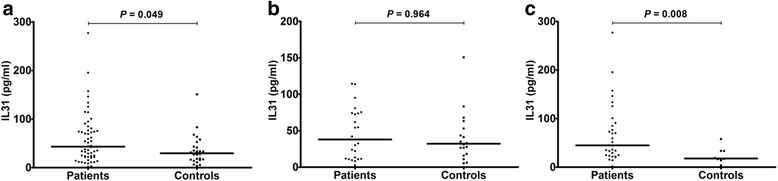
IL-31 serum levels in osteoporotic patients and controls according to age. **a**: IL31 serum levels in all patients and controls; lines represent medians. **b**: IL31 serum levels in patients and controls less than or equal to 65 years; lines represent medians. **c**: IL31 serum levels in patients and controls older than 65 years; lines represent medians

IL-31 serum levels in osteoporotic patients were significantly higher compared to controls (43.12 ± 6.97 pg/ml vs 29.58 ± 6.09 pg/ml; *p* = 0.049) (Fig. 1, panel a). Stratifying patients according to BMD values, no statistically significant differences were found between IL-31 levels in those affected by mild (T-score > -3) compared to severe (T-score < -3) osteoporosis (59.17 ± 9.22 pg/ml vs 37.91 ± 10.52 pg/ml respectively; *p* = 0.876), as well as between patients with mild osteoporosis and controls (*p* = 0.13) and between patients with severe osteoporosis and controls (*p* = 0.059).

Considering the stratification of patients and controls according to age (less than or equal to 65 years and older than 65 years), in subjects under 65, the difference between IL-31 levels in patients (n. 25) compared to controls (n. 18) was not statistically significant (37.91 ± 6.9 pg/ml vs 32.08 ± 8.2 pg/ml; *p* = 0.964) (Fig. 1, panel b), while in older subjects (over 65) IL-31 serum levels were significantly higher in patients (n. 31) compared to controls (n. 8) (45 ± 11.05 pg/ml vs. 17.92 ± 5.92 pg/ml; *p* = 0.008) (Fig. 1, panel c).

In subjects under 65 there was no statistical difference between IL-31 serum levels in patients with mild (n. 13) and severe (n. 12) osteoporosis (42.08 ± 9.86 pg/ml vs. 35.02 ± 10.08 pg/ml; *p* = 0.87), neither between patients with mild osteoporosis and controls (*p* = 0.749) and between patients with severe osteoporosis and controls (*p* = 0.735).

**Fig. 2:**
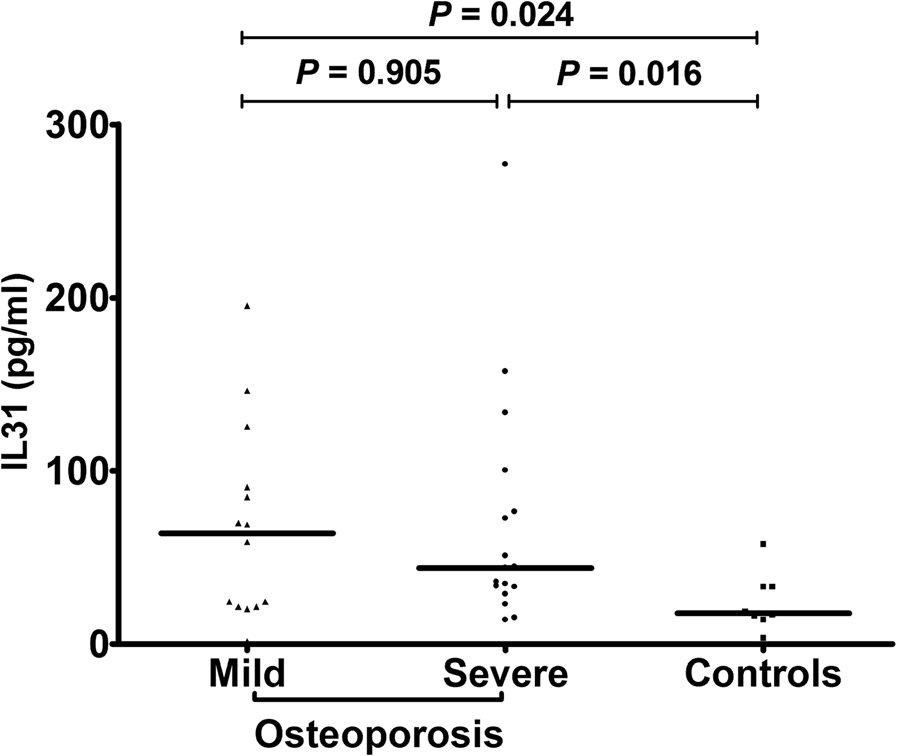
IL31 serum levels in patients divided according to the severity of osteoporosis and controls older than 65 years; lines represent medians

In patients over the age of 65 years with mild osteoporosis (n. 14), IL-31 serum levels were higher (64.17 ± 15.06 pg/ml) compared to age-matched controls (*p* = 0.024), as in patients with severe osteoporosis (n. 17; 44.17 ± 16.29 pg/ml) (*p* = 0.016), whereas no statistically significant difference was found between IL-31 levels in patients over 65 with mild compared to severe osteoporosis (*p* = 0.905) (Fig. 2).

**Fig. 3:**
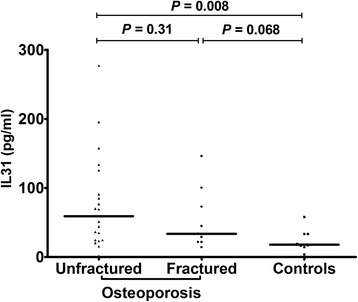
IL31 serum levels in patients divided according to the presence or absence of fragility fractures and controls older than 65 years; lines represent medians

We further divided all patients, according to the presence or absence of fragility fractures, into 2 groups: IL-31 serum levels did not differ significantly between fractured (n. 15) and unfractured (n. 41) osteoporotic women (33.75 ± 9.16 pg/ml and 51.25 ± 8.9 pg/ml; *p* = 0.664), neither between controls and fractured patients (*p* = 0.147), as between controls and unfractured patients (*p* = 0.065). In under 65 years old patients, the levels of IL-31 were not significantly different in fractured (n. 5) compared to unfractured (n. 20) patients (37.9 ± 7.38 pg/ml and 37.29 ± 8.49 pg/ml respectively; *p* = 1). In both fractured and unfractured patients aged less than 65 years, IL-31 serum levels were not significantly different compared to age-matched controls (*p* = 0.602 and *p* = 0.792 respectively). In over 65 patients, there was no difference between IL-31 levels in fractured (n. 10) and unfractured (n. 21) patients (33.54 ± 13.41 pg/ml vs 59.17 ± 14.88 pg/ml; *p* = 0.31). IL-31 serum levels, although higher in over 65 years old patients with fractures compared to age-matched controls, did not reach statistical significance (*p* = 0.068), whereas it was significantly higher in over 65 unfractured patients compared to age-matched controls (*p* = 0.008) (Fig. 3).

Data concerning clinical and anthropometric features of healthy controls and osteoporotic patients are summarized in Table 1. The differences between the two groups were compared by means of the Student’s *t* test for unpaired data and the results are shown in the table. In particular, despite the absence of overt inflammatory pathologies, both ESR and CRP levels were higher in patients compared to controls (19.25 + 8.69 mm/h vs 12 + 7.56 mm/h and 5.75 + 6.6 mg/L vs 2.67 + 3.65 mg/L respectively; *p* < 0.001). Although the osteoporosis group could appear older than controls, the age difference was not statistically significant (*p* = 0.108). Also the BMI of the patients was substantially comparable to that of the controls (26.88 + 4.49 vs 27.70 + 3.33; *p* = 0.415), excluding any bias linked to the influence of this anthropometric parameter on the T-score. Besides the values of T-score (-3.0 + 0.7 vs -0.1 + 1; *p* < 0.001), also BMD values expressed as Z-scores (-1.1 + 0.9 vs 1.4 + 1.1; *p* < 0.001) and g / cm2 (0.701 + 0.07 vs 0.990 + 0.10; *p* < 0.001) are significantly higher in osteoporotic patients than controls.

## Discussion

The major part of recent studies seeking to understand how the immune system impacts and regulates bone remodelling in physiological and pathological conditions through the immunoskeletal interface, have led to a new concept of osteoporosis as a sort of chronic immune mediated disease [1, 2]. Aging and estrogen deficiency are probably the two most important risk factors in developing osteoporosis, although decreased bone mineral density could be related to diverse pathological conditions [4, 6], and in each of these, the influence of very basic determining factors are prevalent rather than others. A possible correlation between plasma levels of certain cytokines and development of osteoporosis has been reported [13, 14]. Significant examples are the elevated production of IFN-γ, IL-6 and IL-17 consequent to an estrogen deficit in menopausal osteoporosis [12, 36] or the action of TNF-α in secondary osteoporosis due to rheumatoid arthritis [14].

In this study, we have demonstrated for the first time an increase of IL-31 serum levels in postmenopausal women with decreased bone mineral density, discovering that there seems to be a correlation between this cytokine and osteoporosis. Serum IL-31 levels did not reflect the severity of osteoporosis, as assessed by BMD values and/or the presence of fractures. The fact that levels of this cytokine don’t correlate neither with the severity of osteoporosis nor with the presence of fractures, appears to exclude the possibility that its increased expression is linked to the role of this cytokine in the progression of the disease. However, the loss of statistical significance between individual subgroups could likely be attributed to the low number of individuals per group and the dispersion of the values of Il-31. The extension of the study to a larger number of patients will better define the involvement of IL-31 in the pathogenesis of osteoporosis.

Moreover, our data clearly showed a relationship between IL-31 expression and osteoporosis in aging, as reflected by the higher levels of this cytokine in aged patients. These findings open the door to a better understanding of the multiple functions of IL-31 in aging as well as in age-related diseases, and provide a new link between bone resorption and IL-31 during aging, suggesting a peculiar role of IL-31 in senile osteoporosis.

IL-31 and its receptors are involved in the regulation of hematopoietic progenitor cell homeostasis and in particular are implicated in the homeostasis of myeloid progenitor cells [17, 21], which are also the common precursors of osteoclasts, the cells specialized in bone resorption [1, 37]. In addition, this cytokine is capable of inducing monocyte activation [17, 38]. It has been documented that IL-31 increases the transcription of proinflammatory genes and induces pro-inflammatory effects in activated human monocytes and macrophages [26].

A variety of cytokines and transcription factors are reported to be involved in the development of osteoporosis, and some of them are regulated by IL-31 [17, 19, 20, 26, 29, 39]. Engagement of the receptor complex results in activation of Janus kinase (JAK) tyrosine kinases and subsequently of different signalling molecules, including different signal transducers and activators of transcription (STAT) factors, Akt, NF-κB [17, 18, 29], as well as the MAPK [29, 30] and PI3K signalling pathways [40]. These pathways are involved in bone remodelling as well as in inflammation [2, 4]. In particular, STAT-1 and STAT-3 proteins are important transcription factors mediating the intracellular events leading to osteoclastic proliferation and activation [41].

Several studies showed that IL-31 stimulated secretion of proinflammatory osteoclastogenic cytokines, chemokines and matrix metalloproteinases [17, 19, 26, 39], all of which are implicated in differentiation, recruitment and function of osteoclasts and are therefore key mediators of bone remodelling during aging [7, 13, 37, 42]. In particular, previous findings have revealed that IL-31 could significantly induce the release of the proinflammatory and osteoclastogenic cytokines IL-1β, IL-6 and chemokines CXCL1, CXCL8, CCL2 and CCL18 from various cell types [17]. These data indicate that IL-31 may function as a proinflammatory cytokine involved in recruitment of osteoclast precursors and in immune mediated bone resorption. The effects of IL-31 are in some way comparable to IL-17A: these two cytokines show additive effects in stimulating the secretion of proinflammatory cytokines and chemokines with bone resorbing capacity [17, 20].

Moreover, proinflammatory cytokines increase, in turn, the expression of IL31 and its receptor complex. The strongest upregulation of IL31, IL31Rα, and OSMR gene expression has been shown after stimulation with IFN‐γ, IL-1β and TNF-α [4, 17, 20, 26]. It has been demonstrated that also reactive oxygen species, which are increased with aging, stimulate the expression of IL-31 in T cells, monocytes and monocyte-derived dendritic cells, inducing inflammatory responses, and this could potentiate age-related bone resorption [39, 43].

The higher levels of CTX and the increase of inflammatory markers in patients compared to controls seem to confirm the presence of a condition of low-grade inflammation and increased bone resorption in osteoporosis. In previous studies, in addition to the increase of inflammation markers, we also documented increased serum levels of well-known proresorptive inflammatory cytokines, such as IL-6 and TNF-α, in osteoporotic patients compared to healthy subjects [44].

Interestingly, IL-17 and senescent memory T cells are the main mediators of bone loss during senescence and inflammaging, that is the progressive establishing of a chronic inflammatory state linked to the continuous long exposure to antigens and ROS during aging [7, 44].

The immunosenescence is characterized by accumulation of memory T cells and exhaustion of naive T cells, as well as by activation of effector inflammatory cells, macrophages and Th17 lymphocytes [44]. Aged osteoporotic subjects displayed significantly higher IL-31 levels with respect to both young and old controls.

It has been proposed that osteoporosis represents a Th1 and Th17 mediated bone inflammation, whereas Th2 cytokines, such as IL-4, display an antiosteoporotic protective function [1, 2, 4, 13–16, 19, 22, 37]. Based on the nature of its main producing cells, IL-31 is considered a Th2 cytokine, its involvement having also been predominantly described firstly in respiratory hypersensitivity and in atopic dermatitis [18, 31]. However IL-31 RNA expression has been reported also in Th1 cells, though higher levels of IL-31 mRNA were observed in Th2 cells [20]. Moreover, its prevalent involvement in the chronic state of atopic dermatitis, were Th1 weighted inflammation is involved, has been documented [28]. Therefore, IL-31 may rather play a role unique to osteoporosis, which represent a chronic condition where Th1 inflammation plays a more dominant role. In addition, in vitro observations indicate that IL-31 can positively and negatively affect Th1 and Th17 differentiation in an APC-free system [17]. These data suggest a potential function for IL-31 in regulating immune responses and inflammation through modulating antigen-presenting cells or more directly T cell themselves and implicate IL-31/IL-31R signalling as a negative regulatory pathway that specifically could limit type 2 inflammation, which is mainly protecting against osteoporosis [45].

Therefore, this study reveals the relationship between IL-31 expression and osteoporosis. However, the precise signal transduction mechanism mediating the effects of IL-31 and its interaction with other cytokines and transcription factors implicated in bone remodelling have to be elucidated. It is likely that additional signals from different cell types modulate IL-31 activity in aged osteoporotic patients. The demonstration of the strong increase of IL-31 in aged osteoporotic subjects may contribute to a better understanding of the mechanisms at the basis of the capacity of the immune system of aged individuals to mount osteoclastogenic immune reactions through peculiar inflammatory responses that may be crucial in senile osteoporosis.

In conclusion, this is the first report on a relationship between IL-31 and bone resorption in aging. Moreover, IL-31 increase that we found in aged osteoporotic patients, probably related to the increased release of this cytokine by senescence inflammatory immune cells, might therefore contribute to the development of a condition of full-blown osteoporosis.

## Conclusion

The increased IL-31 serum levels in aged osteoporotic patients suggests a relationship between bone resorption and overexpression of this cytokine. Elevated IL-31 expression could enhance bone resorption through the induction of chemokines and proinflammatory osteoclastogenic cytokines, which subsequently lead to the recruitment of osteoclast precursors from the bone marrow, their differentiation and activation. Our results suggest that an important role is played by IL-31 in senile osteoporosis. However, how IL-31 acts in concert with known mediators of bone remodelling, remains to be determined. A precise understanding of the involvement of this newly discovered cytokine in the immunopathology of bone resorption is critical to provide more effective strategies for senile osteoporosis treatment.
